# The rising tide of polypharmacy and drug-drug interactions: population database analysis 1995–2010

**DOI:** 10.1186/s12916-015-0322-7

**Published:** 2015-04-07

**Authors:** Bruce Guthrie, Boikanyo Makubate, Virginia Hernandez-Santiago, Tobias Dreischulte

**Affiliations:** Population Health Sciences Division, Medical Research Institute, University of Dundee, Mackenzie Building, Kirsty Semple Way, Dundee, DD2 4BF UK; Department of Public Health, Faculty of Medicine, University of Botswana, Private Bag UB 0022, Gaborone, Botswana; NHS Tayside Medicines Governance Unit, Mackenzie Building, Kirsty Semple Way, Dundee, DD2 4BF UK

**Keywords:** Drug interactions, Family practice, Physician, Polypharmacy, Prescribing patterns, Primary care

## Abstract

**Background:**

The escalating use of prescribed drugs has increasingly raised concerns about polypharmacy. This study aims to examine changes in rates of polypharmacy and potentially serious drug-drug interactions in a stable geographical population between 1995 and 2010.

**Methods:**

This is a repeated cross-sectional analysis of community-dispensed prescribing data for all 310,000 adults resident in the Tayside region of Scotland in 1995 and 2010. The number of drug classes dispensed and the number of potentially serious drug-drug interactions (DDIs) in the previous 84 days were calculated, and age-sex standardised rates in 1995 and 2010 compared. Patient characteristics associated with receipt of ≥10 drugs and with the presence of one or more DDIs were examined using multilevel logistic regression to account for clustering of patients within primary care practices.

**Results:**

Between 1995 and 2010, the proportion of adults dispensed ≥5 drugs doubled to 20.8%, and the proportion dispensed ≥10 tripled to 5.8%. Receipt of ≥10 drugs was strongly associated with increasing age (20–29 years, 0.3%; ≥80 years, 24.0%; adjusted OR, 118.3; 95% CI, 99.5–140.7) but was also independently more common in people living in more deprived areas (adjusted OR most vs. least deprived quintile, 2.36; 95% CI, 2.22–2.51), and in people resident in a care home (adjusted OR, 2.88; 95% CI, 2.65–3.13). The proportion with potentially serious drug-drug interactions more than doubled to 13% of adults in 2010, and the number of drugs dispensed was the characteristic most strongly associated with this (10.9% if dispensed 2–4 drugs vs. 80.8% if dispensed ≥15 drugs; adjusted OR, 26.8; 95% CI 24.5–29.3).

**Conclusions:**

Drug regimens are increasingly complex and potentially harmful, and people with polypharmacy need regular review and prescribing optimisation. Research is needed to better understand the impact of multiple interacting drugs as used in real-world practice and to evaluate the effect of medicine optimisation interventions on quality of life and mortality.

**Electronic supplementary material:**

The online version of this article (doi:10.1186/s12916-015-0322-7) contains supplementary material, which is available to authorized users.

## Background

Prescribed drugs significantly improve a range of health outcomes, but also cause considerable harm. Approximately 6.5% of all emergency hospital admissions are attributable to adverse drug events (ADEs), and at least half of these are judged preventable [1,2]. ADEs have become more common both as a cause of hospital admission [3] and as the reason for outpatient and emergency room visits [4]. ADEs and serious harms occur at all ages, although they are commoner in older people, who are more vulnerable to drug toxicity because of age-related changes in pharmacokinetics and pharmacodynamics, because of multimorbidity and frailty, and because of polypharmacy [4-7]. Polypharmacy is usually defined as concomitant prescription of ≥5 or ≥10 drugs (the latter sometimes called ‘major’ or ‘excessive’ polypharmacy), and there is some evidence of rising rates of polypharmacy, potentially serious drug-drug interactions (DDIs), and ADEs in both outpatient and inpatient settings [3,4,8,9].

The appropriate number of drugs for an individual is highly variable depending on the conditions they have and their functional status, life expectancy, and preferences [10,11]. Among older people taking multiple drugs, there is often evidence of simultaneous over- and under-treatment, with prescribers and patients often struggling to balance benefit and harm in the face of complexity and uncertainty [11]. This partly reflects that evidence of benefit is often derived from trials that usually exclude older adults and people with multimorbidity and do not quantify harms well [12-15], and partly that guidelines often recommend chronic treatments with benefits that are only evident over long periods without explicitly addressing relevance to people with shorter life expectancy [16-18]. Other factors further complicate predicting the benefit/harm of drug treatment, including our limited understanding of ADEs and interactions in people taking large numbers of drugs and the difficulty of distinguishing ADEs from symptoms of existing conditions, risking a ‘prescribing cascade’ where more drugs are used to treat ADEs from existing drugs [6]. Polypharmacy is consistently associated with higher rates of potentially serious DDIs and ADEs, although the increasing use of electronic prescribing with automatic interaction detection might be expected to have reduced this risk over time [4,8,9,19-22]. Although polypharmacy is not always inappropriate, it is frequently problematic, and managing people with multimorbidity and polypharmacy is an important challenge for clinicians and health systems worldwide [5,17]. Of note, although the literature in relation to polypharmacy focuses on older people, polypharmacy is largely driven by multimorbidity and a significant proportion of people with multimorbidity are aged less than 65 years, particularly in the most socioeconomically deprived populations where multimorbidity on average occurs 10 to 15 years earlier than in the most affluent populations [5].

Few studies have used population data to examine changes in polypharmacy and the risk of potentially serious DDIs over time [4,9]. The aim of this study was to use data for all ~310,000 adults resident in a defined geographical area to examine how the prevalence of polypharmacy and potentially serious DDIs changed between 1995 and 2010, and to examine patient and practice characteristics associated with polypharmacy or the presence of a potentially serious DDI in 2010.

## Methods

### Dataset

Prescribing and demographic data were obtained from the University of Dundee Health Informatics Centre (HIC) for all people aged ≥20 years resident in the Tayside region of Scotland for at least 1 year and registered with a National Health Service (NHS) general practitioner (GP) in NHS Tayside on either 31^st^ March 1995 or 31^st^ March 2010. Registration with a single general practice is required to obtain UK NHS care, and with the exception of a few highly specialised drugs, such as biological treatments for inflammatory arthritis, GPs are responsible for all community prescribing to patients. Since the 1990s, HIC has collected data on prescriptions dispensed to Tayside residents by community pharmacies since the 1990s, and these can be linked to each other, using the NHS Scotland unique identifiers (the Community Health Index number) to create a patient-level prescribing record, and to other datasets. For included patients, data on all NHS dispensed prescriptions in the previous 84 days were obtained from a validated research dataset which creates patient-level prescribing records by linking prescriptions using the NHS Scotland unique identifier (the Community Health Index number). We included prescriptions dispensed in the previous 84 days because the most common length of prescription for chronically prescribed drugs is 56 days (usual range 28–84 days), but since patients will not always request repeat prescriptions at precisely the same interval as the prescription length, an 84-day window is the most reliable measure of current exposure. Demographic data available included age, gender, socioeconomic status (measured by the Scottish Index of Multiple Deprivation [23]), and residence in a nursing home (available for 2010 only). Data linkage and anonymization was carried out under HIC Standard Operating Procedures which have been approved by the NHS Research Ethics Service, and all analysis was conducted on anonymised data in the HIC secure Safe Haven. The study was approved by the NHS Tayside Caldicott Guardian, and individual study approval by the NHS Research Ethics Service was therefore not required.

### Defining polypharmacy

We counted the presence of distinct drug classes dispensed in the previous 84 days, which is one of the standard ways of measuring polypharmacy in routine data, giving similar results to other methods [24]. Devices which do not actually deliver drugs (such as blood glucose monitoring equipment), dressings, stoma, or urinary catheter-related products and vaccines were excluded. Drug classes were defined in terms of subsections of the British National Formulary (BNF) [25], which typically contain a single class of agent with similar mechanisms of action (for example, BNF 2.4 corresponds to beta-adrenoreceptor blocking drugs). We expanded the BNF classification where BNF subsections contain multiple drugs which are distinct and commonly co-prescribed (for example, BNF 2.9 antiplatelet drugs was expanded to create BNF 2.9.1 aspirin and BNF 2.9.2 clopidogrel, and so on). The constituents of combination products were separately counted. The complete list of included drug classes is provided in the Additional file 1. We defined three levels of polypharmacy as ≥5, ≥10, and ≥15 drugs dispensed in the previous 84 days.

### Defining potentially serious drug-drug interactions (DDIs)

We examined the frequency of DDIs, defined as co-prescription within the 84-day period on or before 31^st^ March 1995 or 31^st^ March 2010 of pairs of drugs that were listed as having ‘potentially serious’ DDIs (where co-prescription is to be “*avoided or only undertaken with caution and appropriate monitoring*”) in the March 2010 edition of the BNF [25]. In the paper version of the BNF, these interactions are emphasised to prescribers by flagging them with a ‘black dot’, and in the online version by colour coding them red. Of note, we can only measure that a prescription was dispensed, and cannot know whether the patient actually took both drugs simultaneously. However, the measure used is consistent in both years so is reasonable to estimate changes in potential risk. We used the 2010 BNF to define the presence of potentially serious interactions so that the measures were the same in both years.

### Statistical methods

We calculated changes in the number of drugs dispensed and the number of potentially serious interactions experienced, both in total and in terms of the BNF chapter that drugs were listed under. The statistical significance of any differences between 1995 and 2010 were evaluated, using directly age-sex standardised proportions for 2010 to account for the ageing of the population between 1995 and 2010. For 2010, the correlation between patient and general practice characteristics associated with the dispensing of 10 or more drugs and with the presence of any interaction were examined using multilevel modelling to account for the clustering of patients within practices. To assess the extent to which variation in each outcome was attributable to variation between practices, the intra-class correlation co-efficient was estimated in empty models. Data management and analysis were carried out in IBM PASW v21 (IBM Corporation 2012) and multilevel modelling in StataIC v11 (StataCorp 2012). The study was conducted as part of Chief Scientist Office Applied Research Programme Grant ARPG 07/2. The funder had no role in the study design, analysis, or the decision to publish.

## Results

### Study population

There were 301,019 people aged ≥20 years resident in the region in 1995, with a mean age of 48.4 years, rising to 311,811 in 2010, with a mean age of 50.1 years (difference 1.7 years, t = 37.1, *P* <0.001). The proportion of residents aged ≥70 years rose from 15.7% in 1995 to 17.0% in 2010 (difference, 1.3%; 95% confidence interval (CI), 1.1–1.4) but the proportion who were female did not change significantly (51.7% in 1995, 51.5% in 2010, difference 0.2%; 95% CI, 0–0.5).

### Changes in the prevalence of polypharmacy between 1995 and 2010

**Figure 1:**
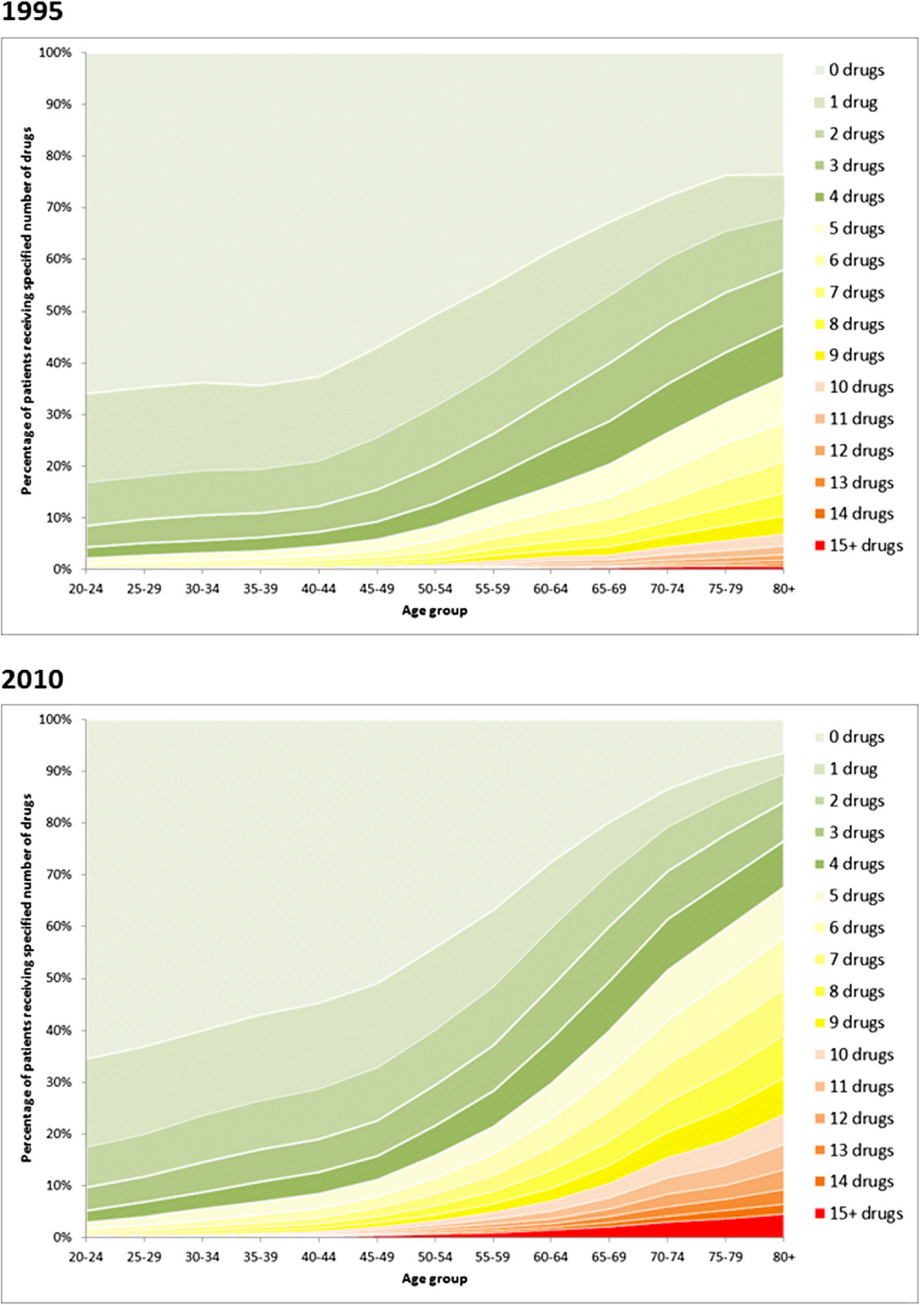
**Number of drug classes dispensed in the 84-day period in 1995 and 2010 by age of patient.**

In 1995, 151,191 (50.6%) people were dispensed one or more drugs in the previous 84 days, compared to 183,726 (58.9%) in 2010 (difference, 8.2%; 95% CI, 8.0–8.4; Table 1), with only a small part of the difference accounted for by population aging (57.8% directly age-sex standardised prevalence in 2010). Between 1995 and 2010, the proportion of people dispensed 5 to 9 drugs rose from 9.7% to 16.3%, dispensed 10 to 14 drugs from 1.5% to 4.7%, and dispensed 15 or more drugs from 0.2% to 1.1% (age-sex standardised relative risks 1.57, 2.92, and 5.58, respectively; Table 1). Drug use in both years was strongly associated with age, with a steady increase in the number of drugs dispensed from early adulthood rising more steeply from middle age (Figure 1). The proportion of people aged 65 and over who were dispensed 10 or more drugs more than tripled between 1995 (4.9%) and 2010 (17.2%).

**Table 1:** **Numbers and class of drugs dispensed to adults in 1995 and 2010**

	**1995**	**2010**	**2010**	
	**No. (%) of patients n = 301,019**	**No. (%) of patients n = 311,881**	Age-sex standardised %*	**Age-sex standardised relative risk 2010 vs. 1995 (95% CI)**
Dispensed 0 drugs	148,828 (49.4)	128,155 (41.1)	42.8	0.87 (0.86–0.87)
Dispensed 1–4 drugs	117,829 (39.1)	114,540 (36.7)	36.4	0.93 (0.92–0.94)
Dispensed 5–9 drugs	29,311 (9.7)	50,972 (16.3)	15.3	1.57 (1.55–1.60)
Dispensed 10–14 drugs	4,481 (1.5)	14,662 (4.7)	4.4	2.92 (2.83–3.02)
Dispensed ≥15 drugs	570 (0.2)	3,552 (1.1)	1.1	5.58 (5.11–6.10)
**Dispensed any drug from BNF chapter**				
1 (gastrointestinal)	37,813 (12.6)	56,536 (18.1)	17.1	1.36 (1.35–1.38)
2 (cardiovascular)	50,593 (16.8)	85,140 (27.3)	25.2	1.49 (1.48–1.51)
3 (respiratory)	18,368 (6.1)	24,760 (7.9)	7.7	1.26 (1.24–1.28)
4 (central nervous system)	55,920 (18.6)	81,902 (26.3)	25.4	1.37 (1.36–1.38)
5 (infections)	48,610 (16.1)	46,934 (15.0)	14.8	0.92 (0.90–0.93)
6 (endocrine)	26,469 (8.8)	44,695 (14.3)	13.5	1.53 (1.51–1.55)
7 (O&G, and urinary tract)	11,695 (3.9)	23,126 (7.4)	7.4	1.91 (1.87–1.95)
8 (malignancy, immunosuppression)	1,686 (0.6)	3,062 (1.0)	0.9	1.64 (1.54–1.74)
9 (nutrition and blood)	8,634 (2.9)	15,217 (4.9)	4.7	1.62 (1.58–1.67)
10 (musculoskeletal)	26,166 (8.7)	26,185 (8.4)	8.0	0.92 (0.91–0.94)
11 (eye)	8,166 (2.7)	11,726 (3.8)	3.5	1.30 (1.27–1.34)
12 (ear, nose, and throat)	8,633 (2.9)	12,459 (4.0)	3.8	1.34 (1.30–1.37)
13 (skin)	23,345 (7.8)	31,644 (10.1)	9.9	1.28 (1.26–1.30)
**Number of BNF chapters dispensed drugs from**				
0	148,828 (49.4)	128,155 (41.1)	42.8	0.87 (0.86–0.87)
1	65,584 (21.8)	64,625 (20.7)	20.7	0.95 (0.94–0.96)
2	40,561 (13.5)	45,439 (14.6)	14.2	1.05 (1.04–1.07)
3	22,548 (7.5)	30,129 (9.7)	9.2	1.23 (1.21–1.25)
4	12,581 (4.2)	19,646 (6.3)	5.9	1.42 (1.39–1.45)
5+	10,917 (3.6)	23,887 (7.7)	7.1	1.97 (1.92–2.01)

*2010 data directly age-sex standardised to 1995 population structure.

### Drug groups associated with changes in polypharmacy between 1995 and 2010

The proportion of people dispensed drugs from each BNF chapter rose significantly for every chapter except drugs for infections and musculoskeletal drugs, where use fell slightly (Table 1). The largest absolute rises were for cardiovascular (27.3% of the population in 2010 vs. 16.8% in 1995; age-sex standardised relative risk [sRR], 1.49), central nervous system (26.3% vs. 18.6%; sRR, 1.37), gastrointestinal (18.1% vs. 12.6%; sRR, 1.36), and endocrine drugs (14.3% vs. 8.8%, sRR, 1.53). The number of BNF chapters that patients were dispensed drugs from increased, with 10,917 (3.6%) patients dispensed drugs from five or more chapters in 1995 compared to 23,887 (7.7%) in 2010 (sRR, 1.97).

### Patient and practice characteristics associated with polypharmacy in 2010

In multilevel modelling, age was the patient characteristic most strongly associated with dispensing of ≥10 drugs rising from 0.3% of those aged 20 to 29 years to 22.9% of those aged ≥80 (adjusted OR [aOR], 118.3; 95% CI, 99.5–140.7) (Table 2). People living in care homes were much more likely to be dispensed ≥10 drugs in univariate analysis (36.5% vs. 5.5% of those living at home), although the association between care home residency and polypharmacy was greatly reduced in the adjusted model reflecting that care home residents are much older than average (aOR, 2.88; 95% CI, 2.65–3.13). After adjustment, people living in more deprived areas had over twice the odds of being dispensed ≥10 drugs. Women were slightly more likely to have polypharmacy than men, as were people living in urban compared to more rural areas. Variation between practices was modest, with an intra-class correlation coefficient of 0.009 in the empty model (interpretable as 0.9% of the variation in outcomes being due to variation between practices). None of the practice characteristics examined were significantly associated with dispensing of ≥10 drugs.

**Table 2:** Patient characteristics associated with dispensing of ≥10 drugs in 2010*

**Variable (no. of patients)**	**Proportion (95% CI) dispensed ≥10 drugs**	**Univariate multilevel odds ratio (95% CI)**	**Adjusted multilevel odds ratio (95% CI)**
**Age groups (years)**			
20–29 (n = 51,197)	0.3 (0.2–0.3)	Reference	Reference
30–39 (n = 47,857)	0.7 (0.6–0.8)	2.81 (2.31–3.41)	2.91 (2.38–3.56)
40–49 (n = 60,077)	1.7 (1.5–2.0)	6.78 (5.69–8.09)	7.16 (5.98–8.58)
50–59 (n = 52,751)	4.1 (3.6–4.6)	16.9 (14.2–20.0)	18.0 (15.1–21.5)
60–69 (n = 47,080)	8.7 (7.9–9.4)	38.1 (32.2–45.2)	41.1 (34.6–48.8)
70–79 (n = 32,986)	17.1 (16.1–18.2)	83.4 (70.5–98.7)	87.5 (73.7–104.0)
80+ (n = 19,933)	24.0 (22.9–25.1)	130.0 (10.9.8–153.9)	118.3 (99.5–140.7)
**Sex**			
Male (n = 151,202)	4.6 (4.3–4.8)	Reference	Reference
Female (n = 160,679)	7.0 (6.7–7.4)	1.59 (1.54–1.64)	1.34 (1.30–1.39)
**Deprivation quintile**			
1 (affluent) (n = 56,416)	4.9 (4.5–5.4)	Reference	Reference
2 (n = 94,090)	5.3 (5.0–5.7)	1.13 (1.07–1.19)	1.21 (1.14–1.28)
3 (n = 53,990)	5.3 (5.0–5.6)	1.04 (0.99–1.10)	1.32 (1.24–1.39)
4 (n = 49,387)	6.4 (6.0–6.7)	1.33 (1.26–1.40)	1.70 (1.61–1.79)
5 (deprived) (n = 50,877)	7.6 (7.1–8.1)	1.58 (1.49–1.67)	2.36 (2.22–2.51)
**SEURC category** ^**#**^			
Primary city (n = 121,804)	6.5 (6.1–6.9)	Reference	Reference
Urban area (n = 80,401)	6.0 (5.5–6.5)	0.91 (0.84–1.00)	0.96 (0.88–1.05)
Accessible area (n = 78,490)	5.0 (4.6–5.4)	0.74 (0.69–0.80)	0.84 (0.78–0.92)
Remote area (n = 24,268)	5.7 (5.0–6.3)	0.85 (0.75–0.96)	0.83 (0.73–0.94)
**Living**			
In own home (n = 308,660)	5.5 (5.2–5.8)	Reference	Reference
In care home (n = 3,221)	36.5 (33.8–39.1)	9.91 (9.21–10.67)	2.88 (2.65–3.13)

*Practice level variables not significant in either univariate or adjusted models (list size, accreditation for postgraduate training, dispensing medicines [marginally significant association in univariate analysis], type of NHS contract).

^#^Scottish Executive Urban–rural Classification.

### Changes in potentially serious DDIs between 1995 and 2010

**Figure 2:**
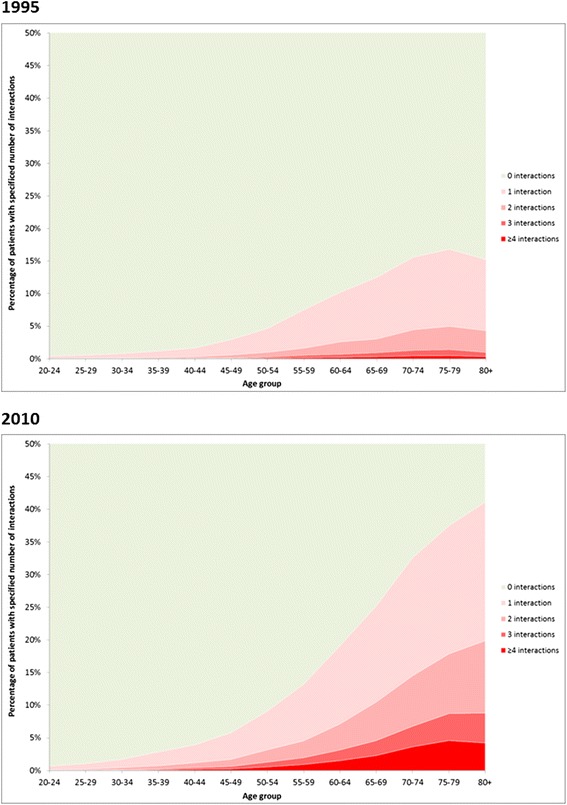
**Number of potentially serious drug-drug interactions in the 84-day period in 1995 and 2010 by age of patients.**

Potentially serious DDIs were over twice as common in 2010 than 1995, with 5.8% of adults in 1995 having at least one DDI compared to 13.1% in 2010 (sRR, 2.08; 95% CI, 2.04–2.12; Table 3). There were larger relative increases in the proportion of the population with multiple potentially serious DDIs; for example, the proportion with ≥2 DDIs more than tripling from 1.5% of adults in 1995 to 5.6% in 2010. In both years, older people were more likely to have a potentially serious DDI (Figure 2, Table 4), with 10,272 (15.2%) of people aged ≥65 having at least one in 1995 compared to 25,071 (34.1%) in 2010.

**Table 3:** **Potentially serious drug-drug interactions in 1995 and 2010**

	**1995**	**2010**	**2010**	
	**No. (%) of patients**	**No. (%) of patients**	Age-sex standardised % of patients*	**Age-sex standardised relative risk 2010 vs. 1995 (95% CI)**
	**n = 301,019**	**n = 311,881**	**n = 311,881**	
**All patients**	17,448 (5.8)	40,689 (13.0)	12.1	2.08 (2.04–2.12)
**No. of interactions**				
0	283,571 (94.2)	271,192 (86.9)	87.9	0.91 (0.91–0.91)
1	13,051 (4.3)	23,907 (7.7)	7.1	2.41 (2.35–2.47)
2	3,151 (1.0)	9,324 (3.0)	2.8	5.54 (5.25–5.85)
3	814 (0.3)	3,776 (1.2)	1.1	7.03 (6.39–7.74)
4+	432 (0.1)	3,682 (1.2)	1.1	13.8 (12.1–15.7)
**Any interaction involving drugs from BNF chapter**				
1 (gastrointestinal)	197 (0.07)	1,452 (0.47)	0.42	6.30 (5.44–7.31)
2 (cardiovascular)	14,236 (4.7)	34,124 (10.9)	9.9	2.09 (2.05–2.13)
3 (respiratory)	863 (0.30)	863 (0.28)	0.26	0.84 (0.77–0.93)
4 (central nervous system)	3,489 (1.2)	11,465 (3.7)	3.4	2.93 (2.81–3.04)
5 (infections)	1,062 (0.37)	1,526 (0.49)	0.45	1.22 (1.13–1.32)
6 (endocrine)	688 (0.23)	1,588 (0.51)	0.47	2.00 (1.83–2.19)
7 (O&G, and urinary tract)	257 (0.09)	2,516 (0.81)	0.68	7.66 (6.75–8.69)
8 (malignancy, immunosuppression)	125 (0.04)	207 (0.07)	0.06	1.44 (1.15–1.79)
9 (nutrition and blood)	252 (0.09)	62 (0.02)	0.02	0.22 (0.16–0.29)
10 (musculoskeletal)	2,565 (0.87)	4,717 (1.5)	1.4	1.64 (1.56–1.72)
11 (eye)	21 (0.01)	9 (0.003)	0.003	0.38 (0.17–0.82)
12 (ear, nose, and throat)	0	0	0	–
13 (skin)	0	0	0	–

*2010 data directly age-sex standardised to 1995 population structure.

**Table 4:** **Patient characteristics associated with the presence of a potentially serious drug-drug interaction for people dispensed at least two drugs in 2010**

**Variable (no. of patients)**	**Proportion (95% CI) with any DDI**	**Univariate multilevel odds ratio (95% CI)**	**Adjusted multilevel odds ratio (95% CI)**
**Age groups (years)**			
20–29 (n = 9,976)	4.5 (4.0–5.0)	Reference	Reference
30–39 (n = 12,294)	9.2 (8.6–9.8)	2.18 (1.5–2.44)	1.88 (1.68–2.11)
40–49 (n = 18,805)	15.8 (14.9–16.6)	4.05 (3.65–4.49)	3.05 (2.75–3.39)
50–59 (n = 23,565)	25.1 (24.0–26.2)	7.28 (6.59–8.04)	4.67 (4.21–5.17)
60–69 (n = 30,756)	33.8 (32.8–34.8)	11.2 (10.1–12.3)	6.05 (5.47–6.69)
70–79 (n = 27,240)	42.5 (41.4–43.6)	16.2 (14.7–17.9)	6.98 (6.31–7.72)
80+ (n = 17,977)	46.0 (44.8–47.1)	18.8 (17.0–20.7)	7.34 (6.62–8.14)
**Sex**			
Male (n = 58,466)	30.7 (30.0–31.5)	Reference	Reference
Female (n = 82,147)	27.7 (27.0–28.4)	0.87 (0.85–0.88)	0.85 (0.83–0.88)
**Place of residence**			
Living in own home (n = 13,615)	28.7 (28.1–29.4)	Reference	Reference
Living in care home (n = 2,998)	38.0 (36.3–39.7)	1.51 (1.40–1.63)	0.51 (0.47–0.56)
**No. of drugs dispensed in last 84 days**			
2–4 (n = 71,427)	10.9 (10.4–11.4)	Reference	Reference
5–9 (n = 50,972)	40.0 (39.0–40.9)	5.49 (5.33–5.65)	4.39 (4.26–4.53)
10–14 (n = 14,662)	65.9 (64.9–67.0)	16.1 (15.5–16.8)	12.0 (11.5–12.5)
15+ (n = 3,552)	80.8 (79.4–82.2)	35.3 (32.3–38.5)	26.8 (24.5–29.3)

Patient level socioeconomic deprivation and urban/rural residence, and practice level variables (list size, accreditation for postgraduate training, dispensing medicines, type of NHS contract) were not significant in either univariate or adjusted models and are not shown.

### Drug groups associated with changes in potentially serious DDIs between 1995 and 2010

Table 3 shows that the drug groups most commonly implicated in potentially serious DDIs in 1995 were cardiovascular (affecting 4.7% of adults), central nervous system (1.2%), and musculoskeletal (0.9%) drugs. These remained the three drug groups most implicated in 2010, but with significantly increased prevalence (10.9%, 3.7%, and 1.5%, respectively; sRR, 2.09, 2.93, and 1.64). There were larger relative but smaller absolute increases in interactions associated with obstetric and gynaecological drugs (from 0.09% to 0.81%; sRR, 7.66) and gastrointestinal drugs (from 0.07% to 0.47%; sRR, 6.30). Other drug groups changed more variably, although absolute rates were low in 1995 and absolute differences between years were much smaller.

### Patient and practice characteristics associated with DDIs in 2010

In multilevel modelling, the number of drugs dispensed was the characteristic most strongly associated with the presence of a potentially serious DDI among people dispensed at least 2 drugs (the minimum number required for a DDI to be present). The proportion of people with potentially serious DDIs was 80.8% among those dispensed ≥15 drugs compared to 10.9% of those dispensed 2 to 4 drugs (aOR, 26.8; 95% CI, 24.5–29.3). Older people were also much more likely to be prescribed drugs with an interaction, as were men to a small extent. The strength of the association between older age and presence of DDIs was significantly weakened after adjustment for numbers of drugs dispensed (reflecting much higher rates of drug use in the elderly), but people aged 80 and over were still much more likely to have DDIs, with an adjusted odds ratio of 7.34 (95% CI, 6.62–8.14) compared to 20 to 29 year olds. In contrast, after adjustment for age and the number of drugs dispensed, people in care homes were less likely to be dispensed interacting drugs. There was more variation between practices for prescribing drugs with potentially serious interactions than there was for polypharmacy, with an intra-class correlation coefficient in the empty model of 0.031. However, none of the practice characteristics examined were significantly associated with potentially serious DDIs.

## Discussion

Between 1995 and 2010 there were large increases in the number of patients with polypharmacy due to an increase in the use of drugs from all but two BNF chapters. The dispensing of ≥5 drugs increased from 11.4% to 20.8% of adults, and the dispensing of ≥10 drugs increased from 1.7% to 5.8%. Receipt of ≥10 drugs was very strongly associated with increasing age, but was also independently more common in women, in people living in more deprived areas, and in care home residents. Associated with this, the proportion of adults with potentially serious DDIs more than doubled, with 13.0% of adults in 2010 being dispensed a combination of drugs with the potential to cause serious harm. Interactions increased in prevalence dramatically with the number of drugs dispensed, rising from 10.9% in those dispensed 2 to 4 drugs to 80.8% dispensed 15 or more in 2010. Age was also strongly associated with being dispensed interacting drugs, and this association persisted (albeit weaker) after adjusting for the number of drugs an individual was dispensed. Interestingly, although men were less likely to be prescribed large numbers of drugs, they were more likely to be dispensed interacting drugs.

These population-based results are similar to the limited published research in more selective populations. In a representative survey of adults aged ≥77 years in Sweden, Haider et al. showed that the percentage prescribed ≥5 drugs rose from 18% in 1992 to 42% in 2002, with an increase from 17% to 25% of older people exposed to a potentially serious DDI [9]. Between 2000 and 2010 in Italy, the proportion of people aged ≥65 years prescribed five or more active agents rose from 43% to 53%, with larger rises in those aged ≥85 years [26]. In the USA, the proportion of outpatient consultations in which patients were taking 5 or more medications rose from 6% to 15% between 1995 and 2005, and the rate of outpatient or emergency room consultations where an ADE was reported rose from 13.2 per 1,000 persons to 18.1 per 1,000 persons, with ADE rates increasing with the number of drugs a patient was taking [4]. Swedish and Italian data also show a strong relationship between numbers of drugs dispensed and potentially serious DDIs [8,19].

A key strength of the study is the use of dispensed prescribing data for a defined geographical population collected using the same method, but all data of this sort has several limitations. Over-the-counter sales are not accounted for, which is important for drugs such as analgesics (paracetamol, selected non-steroidal anti-inflammatory drugs, and low dose codeine), simple antacids, and antihistamines, as well as non-prescribed products with potential for interactions such as St John’s Wort. Actual drug use and interactions will therefore be underestimated in both years. In contrast, interactions are likely to be somewhat overestimated by our counting co-prescription in an 84-day period as indicating the presence of an interaction since, in some cases, patients will have stopped one drug before starting another. However, the measure is consistent between the two years and is therefore a reasonable estimate of the scale of the change in risk. The interactions being counted are also potentially, rather than always, harmful, and most interactions will not cause harm, although much harm is also unrecognised by clinicians. However, since measurement is consistent over the period examined, we believe that the patterns of change seen will plausibly reflect changes in harm as well. The main interaction comparison also applies 2010 knowledge to both years, which effectively penalises prescribers in 1995 since the measure includes interactions which they could not be aware of. However, this is likely to make the change observed a conservative estimate and it is worth noting that the estimate of the potential interaction rate in 2010 is itself an underestimate since knowledge continues to evolve (a recent example being the 2012 UK regulatory risk communication concerning dose restrictions for simvastatin when co-prescribed with calcium-channel antagonists, which on its own would have affected 2.4% of Tayside adults in 2010) [27].

## Conclusions

Although this study cannot identify which is most important, there are several reasons why rates of prescribing are likely to have risen, including the greater availability of effective drugs, the promotion of consistent treatment of many chronic conditions by guidelines and other quality improvement interventions, and changes in patient expectations. Of particular note is that guidelines increasingly recommend multiple drug therapy to achieve tight intermediate outcomes such as blood pressure and glycaemic control. This highlights that polypharmacy is potentially problematic rather than always inappropriate, because potentially serious interactions do not always cause harm or may be a price worth paying for benefits. However, most evidence of effectiveness is from randomised trials which usually exclude older people and those with multimorbidity and polypharmacy [17]. Our understanding of the benefits of real-world complex treatment regimens is therefore limited, and our understanding of harms largely restricted to pairwise interactions rather than the often more complex reality.

The key clinical implication is that clinicians need to regularly review and optimise chronic medication, particularly in people with polypharmacy or whose life expectancy is limited to a few years in whom drugs for prevention are less likely to be beneficial. Since polypharmacy almost inevitably involves drugs for multiple conditions, medication reviews will typically have to be the responsibility of generalist physicians or pharmacists, who require appropriate training in how to personalise treatment in people with multimorbidity or frailty in order to minimise harm while retaining drugs with large benefit [5]. Clinical guidelines also need to consider making recommendations on when drugs should be stopped, although the lack of discussion in guidelines about treatment cessation at least partly reflects the lack of evidence in this area [16-18]. There is therefore a need for research to evaluate the impact of treatment cessation in frailer people with shorter life expectancy, focusing on drugs with small prognostic benefits that accrue over long periods of time. Further research is needed to improve our understanding of the risks and benefits of the complex combinations of potentially interacting drugs that happen in the real world, and for trials of interventions to optimise treatment of people with polypharmacy, which evaluate the impact on mortality and quality of life as well as prescribing outcomes [28].

## Additional file

Additional file 1:
**The rising tide of polypharmacy and drug-drug interactions: repeated cross-sectional population analysis 1995-2010.**

## Abbreviations

ADEs: Adverse drug events
aOR: Adjusted odds ratio
BNF: British National Formulary
CI: Confidence intervals
DDIs: Drug-drug interactions
GP: General practitioner
HIC: Health Informatics Centre
NHS: National Health Service
sRR: Standardised relative risk
