# Supplementary material for: The rising tide of polypharmacy and drug-drug interactions: population database analysis 1995–2010
Source: BMC Med. 2015 Apr 7;13:74. doi: 10.1186/s12916-015-0322-7 (PMC4417329; doi:10.1186/s12916-015-0322-7)
Supplement: Additional file 1: — The rising tide of polypharmacy and drug-drug interactions: repeated cross-sectional population analysis 1995-2010. [file 12916_2015_322_MOESM1_ESM.doc]

### Supplementary file: The rising tide of polypharmacy and drug-drug interactions: repeated cross-sectional population analysis 1995-2010

Table S1: Drug classes included in the polypharmacy count (where drugs in the same BNF subsection are commonly co-prescribed, then they are listed and counted separately)

| **Drug class name** | **BNF subsection** |
| --- | --- |
| **Chapter 1 Gastro-intestinal system** |  |
| Antacids and simeticone | 1.1.1 |
| Compound alginates and proprietary antacids | 1.1.2 |
| Antispasmodics & drugs altering gut motility – antimuscarinics | 1.2 |
| Antispasmodics & drugs altering gut motility - other antispasmodics | 1.2 |
| Antispasmodics and drugs altering gut motility – cisapride | 1.2 |
| H_2_ receptor antagonists | 1.3.1 |
| Selective antimuscarinics | 1.3.2 |
| Chelates and complexes | 1.3.3 |
| Prostaglandin analogues | 1.3.4 |
| Proton pump inhibitors | 1.3.5 |
| Liquorice | 1.3.6 |
| Adsorbents and bulk forming drugs | 1.4.1 |
| Antimotility drugs | 1.4.2 |
| Aminosalicylates | 1.5.1 |
| Corticosteroids for chronic bowel disorders | 1.5.2 |
| Gut drugs affecting immune response | 1.5.3 |
| Food allergy | 1.5.4 |
| Bulk forming laxatives | 1.6.1 |
| Stimulant laxatives | 1.6.2 |
| Faecal softeners | 1.6.3 |
| Osmotic laxatives | 1.6.4 |
| Peripheral opioid-receptor antagonists | 1.6.6 |
| 5HT4 receptor antagonists | 1.6.7 |
| Local preparations for anal and rectal disorders | 1.7 |
| Drugs affecting biliary composition and flow | 1.9.1 |
| Bile acid sequestrants | 1.9.2 |
| Pancreatin | 1.9.4 |
| **Chapter 2 – Cardiovascular system** |  |
| Cardiac glycosides | 2.1.1 |
| Phospodiesterase inhibitors | 2.1.2 |
| Thiazides and related diuretics | 2.2.1 |
| Loop diuretics | 2.2.2 |
| Potassium sparing diuretics | 2.2.3 |
| Aldosterone antagonists | 2.2.3 |
| Osmotic diuretics | 2.2.5 |
| Mercurial diuretics | 2.2.6 |
| Carbonic anydrase inhibitors | 2.2.7 |
| Anti-arrhythmic drugs | 2.3 |
| Beta-adrenoreceptor blocking drugs | 2.4 |
| Vasodilator antihypertensive drugs | 2.5.1 |
| Centrally acting antihypertensive drugs | 2.5.2 |
| Adrenergic neurone blocking drugs | 2.5.3 |
| Alpha-adrenoreceptor blocking drugs | 2.5.4 |
| ACE inhibitors | 2.5.5.1 |
| Angiotensin-II receptor antagonists | 2.5.5.2 |
| Renin inhibitors | 2.5.5.3 |
| Nitrates | 2.6.1 |
| Calcium channel blockers | 2.6.2 |
| Other antianginal drugs | 2.6.3 |
| Peripheral vasodilators and related drugs | 2.6.4 |
| Inotropic sympathomimetics | 2.7.1 |
| Vasoconstrictor sympathomimetics | 2.7.2 |
| Parenteral anticoagulants | 2.8.1 |
| Oral anticoagulants | 2.8.2 |
| Aspirin | 2.9 |
| Clopidogrel | 2.9 |
| Dipyridamole | 2.9 |
| Ticlodipine | 2.9 |
| Other antiplatelet drugs | 2.9 |
| Antifibrinolytic drugs and haemostatics | 2.11 |
| Statins | 2.12 |
| Bile acid sequestrants | 2.12 |
| Ezetimibe | 2.12 |
| Fibrates | 2.12 |
| Nicotinic acid group | 2.12 |
| Omega-3 fatty acid compounts | 2.12 |
| Other lipid lowering | 2.12 |
| **Chapter 3 Respiratory system** |  |
| Short acting beta2 adrenoreceptor agonists | 3.1.1.1 |
| Long acting beta2 adrenoreceptor agonists | 3.1.1.1 |
| Other adrenoreceptor agonists | 3.1.1.2 |
| Antimuscarinic bronchodilators | 3.1.2 |
| Theophylline | 3.1.3 |
| Inhaled corticosteroids | 3.2 |
| Cromoglicate and related therapy | 3.3 |
| Leukotriene receptor antagonists | 3.3 |
| Antihistamines non-sedating | 3.4.1 |
| Antihistamines sedating | 3.4.1 |
| Antihistamines unknown sedation | 3.4.1 |
| Allergic emergencies | 3.4.3 |
| Mucolytics | 3.7 |
| Cough suppressants | 3.9 |
| Systemic nasal decongestants | 3.10 |
| **Chapter 4 Central nervous system** |  |
| Hypnotic benzodiazepines | 4.1.1 |
| Hypnotic z-drugs | 4.1.1 |
| Hypnotic chloral and derivatives | 4.1.1 |
| Hypnotic chlormethiazole | 4.1.1 |
| Hypnotic sodium oxybate | 4.1.1 |
| Hypnotic melatonin | 4.1.1 |
| Hypnotic other | 4.1.1 |
| Anxiolytic benzodiazepines | 4.1.2 |
| Anxiolytic buspirone | 4.1.2 |
| Anxiolytic meprobamate | 4.1.2 |
| Anxiolytic other | 4.1.2 |
| Barbiturates | 4.1.3 |
| Typical antipsychotics | 4.2.1 |
| Atypical antispsychotics | 4.2.1 |
| Depot antipsychotics | 4.2.2 |
| Antimanic valproic acid | 4.2.3 |
| Antimanic lithium | 4.2.3 |
| Tricylic antidepressants | 4.3.1 |
| Tricyclic related antidepressants | 4.3.1 |
| MAOI antidepressants | 4.3.2 |
| SSRI antidepressants | 4.3.3 |
| Other antidepressants | 4.3.4 |
| CNS stimulants and drugs used in ADHD | 4.4 |
| Anti-obesity drugs acting on GI tract | 4.5.1 |
| Centrally acting appetite suppressants | 4.5.2 |
| Nausea antihistamines | 4.6 |
| Nausea phenothiazines | 4.6 |
| Nausea domperidone/metoclopramide | 4.6 |
| Nausea 5HT3 antagonists | 4.6 |
| Nausea neurokinin receptor antagonist | 4.6 |
| Nausea cannabinoid | 4.6 |
| Nausea hyoscine | 4.6 |
| Nausea betahistine | 4.6 |
| Nausea other | 4.6 |
| Non-opioid analgesics paracetamol | 4.7.1 |
| Non-opioid analgesics aspirin | 4.7.1 |
| Non-opioid analgesics nefopam | 4.7.1 |
| Non-opioid analgesics other | 4.7.1 |
| Opioid analgesics | 4.7.2 |
| Treatment of acute migraine | 4.7.4.1 |
| Prophylaxis of migraine | 4.7.4.2 |
| Cluster headache and the trigeminal autonomic cephalgias | 4.7.4.3 |
| Epilepsy carbamazepine | 4.8.1 |
| Epilepsy eslicarbazepine | 4.8.1 |
| Epilepsy oxcarbazepine | 4.8.1 |
| Epilepsy ethosuximide | 4.8.1 |
| Epilepsy gabapentin | 4.8.1 |
| Epilepsy pregabalin | 4.8.1 |
| Epilepsy lacosamide | 4.8.1 |
| Epilepsy lamotrigine | 4.8.1 |
| Epilepsy levetiracetam | 4.8.1 |
| Epilepsy barbiturates | 4.8.1 |
| Epilepsy phenytoin | 4.8.1 |
| Epilepsy rufinamide | 4.8.1 |
| Epilepsy tiagabine | 4.8.1 |
| Epilepsy topiramate | 4.8.1 |
| Epilepsy sodium valproate | 4.8.1 |
| Epilepsy vigabatrin | 4.8.1 |
| Epilepsy zonisamide | 4.8.1 |
| Epilepsy benzodiazepines | 4.8.1 |
| Drugs used in status epilepticus | 4.8.2 |
| Parkinsons dopamine receptor agonists | 4.9.1 |
| Parkinsons levodopa | 4.9.1 |
| Parkinsons MAOI-B inhibitors | 4.9.1 |
| Parkinsons catechol-o-methyltransferase inhibitors | 4.9.1 |
| Parkinsons amantadine | 4.9.1 |
| Antimuscarinic drugs used in Parkinsons Disease | 4.9.2 |
| Drugs used in essential tremor, chorea, tics and related disorders | 4.9.3 |
| Alcohol dependence | 4.10 |
| Cigarettes dependence | 4.10 |
| Opioid dependence | 4.10 |
| Drugs for dementia | 4.11 |
| **Chapter 5 Infections** |  |
| Benzylpenicillin and pen V | 5.1.1.1 |
| Penicillinase resistant pencillins | 5.1.1.2 |
| Broad spectrum penicillins | 5.1.1.3 |
| Antipseudomonal penicillins | 5.1.1.4 |
| Mecillinams | 5.1.1.5 |
| Cephalosporins | 5.1.2.1 |
| Carbapenems | 5.1.2.2 |
| Other beta-lactams | 5.1.2.3 |
| Tetracyclines | 5.1.3 |
| Aminoglycosides | 5.1.4 |
| Macrolides | 5.1.5 |
| Clindamycin | 5.1.6 |
| Other antibacterials | 5.1.7 |
| Sulphonamides | 5.1.8 |
| Trimethoprim | 5.1.8 |
| TB capreomycin | 5.1.9 |
| TB cycloserine | 5.1.9 |
| TB ethambutol | 5.1.9 |
| TB isoniazid | 5.1.9 |
| TB pyrazinamide | 5.1.9 |
| TB rifabutin | 5.1.9 |
| TB rifampicin | 5.1.9 |
| TB streptomycin | 5.1.9 |
| Antileprotic drugs | 5.1.10 |
| Metronidazole & tinidazole | 5.1.11 |
| Quinolones | 5.1.12 |
| Nitrofurantoin | 5.1.13 |
| Triazole antifungals | 5.2.1 |
| Imidazole antifungals | 5.2.2 |
| Polyene antifungals | 5.2.3 |
| Echinocandin antifungals | 5.2.4 |
| Other antifungals | 5.2.5 |
| HIV abacavir | 5.3.1 |
| HIV didanosine | 5.3.1 |
| HIV emtricitabine | 5.3.1 |
| HIV lamivudine | 5.3.1 |
| HIV stavudine | 5.3.1 |
| HIV tenofovir | 5.3.1 |
| HIV zidovudine | 5.3.1 |
| HIV atazanavir | 5.3.1 |
| HIV darunavir | 5.3.1 |
| HIV fosamprenavir | 5.3.1 |
| HIV indinavir | 5.3.1 |
| HIV lopinavir | 5.3.1 |
| HIV nelfinavir | 5.3.1 |
| HIV ritonavir | 5.3.1 |
| HIV saquinavir | 5.3.1 |
| HIV tipranavir | 5.3.1 |
| HIV efavirenz | 5.3.1 |
| HIV etravirine | 5.3.1 |
| HIV nevirapine | 5.3.1 |
| HIV enfuvirtide | 5.3.1 |
| HIV maraviroc | 5.3.1 |
| HIV raltegravir | 5.3.1 |
| HIV zalcetabine | 5.3.1 |
| Herpes simplex and varicella-zoster infection | 5.3.2.1 |
| Cytomegalovirus infection | 5.3.2.2 |
| Viral hepatitis | 5.3.3 |
| Influenza | 5.3.4 |
| Respiratory syncytial virus | 5.3.5 |
| Antimalarials | 5.4.1 |
| Amoebicides | 5.4.2 |
| Antigiardial drugs | 5.4.4 |
| Leishmaniacides | 5.4.5 |
| Trypanocides | 5.4.6 |
| Drugs for toxoplasmosis | 5.4.7 |
| Drugs for pneumocystic pneumonia | 5.4.8 |
| Drugs for threadworms | 5.5.1 |
| Ascaricides | 5.5.2 |
| Drugs for tapeworm infections | 5.5.3 |
| Drugs for hookworms | 5.5.4 |
| Schistomicides | 5.5.5 |
| Filaricides | 5.5.6 |
| Drugs for cutaneous larva migrans | 5.5.7 |
| Drugs for strongyloides | 5.5.8 |
| **Chapter 6 Endocrine system** |  |
| Insulins | 6.1.1 |
| Sulphonylyureas | 6.1.2.1 |
| Biguanides | 6.1.2.2 |
| Thiazolidinediones | 6.1.2.3 |
| Glinides | 6.1.2.3 |
| Gliptins | 6.1.2.3 |
| Exenatide and liraglutide | 6.1.2.3 |
| Acarbose and guar gum | 6.1.2.3 |
| Thyroid hormones | 6.2.1 |
| Antithyroid drugs | 6.2.2 |
| Replacement corticosteroids | 6.3.1 |
| Treatment corticosteroids | 6.3.2 |
| Oestrogens and HRT | 6.4.1.1 |
| Progestogens | 6.4.1.2 |
| Male sex hormones and antagonists | 6.4.2 |
| Anabolic steroids | 6.4.3 |
| Hypothalamic & anterior pituitary hormones & anti-oestrogens | 6.5.1 |
| Posterior pituitary hormones and antagonists | 6.5.2 |
| Calcitonin and parathyroid hormone | 6.6.1 |
| Bisphosphonates and other drugs affecting bone metabolism | 6.6.2 |
| Bromocriptine and other dopaminergic drugs | 6.7.1 |
| Drugs affecting gonadotrophins | 6.7.2 |
| Metyrapone and trilostane | 6.7.3 |
| Somatomedins | 6.7.4 |
| **Chapter 7 Obstetrics, gynaecology and urinary tract disorders** |  |
| Topical hormone replacement therapy | 7.2.1 |
| Vaginal and vulval infections | 7.2.2 |
| Combined oral contraceptives | 7.3.1 |
| Oral progestogen only contraceptives | 7.3.2.1 |
| Parenteral progestogen only contraceptives | 7.3.2.2 |
| Emergency contraception | 7.3.5 |
| Urinary retention - alpha blockers | 7.4.1 |
| Urinary retention – parasympathomimetics | 7.4.1 |
| Drugs for urinary frequency, enuresis, incontinence | 7.4.2 |
| Drugs used in urological pain | 7.4.3 |
| Drugs for erectile dysfunction | 7.4.5 |
| **Chapter 8 Malignant disease and immunosuppression** |  |
| Alkylating drugs | 8.1.1 |
| Anthracylcines and other cytotoxic antibiotics | 8.1.2 |
| Antimetabolites | 8.1.3 |
| Vinca alkaloids | 8.1.4 |
| Other antineoplastic | 8.1.5 |
| Antiproliferative azathioprine | 8.2.1 |
| Antiproliferative mycophenolate | 8.2.1 |
| Other immunosuppressants ciclosporin | 8.2.2 |
| Other immunosuppressants sirolimus/tacrolimus | 8.2.2 |
| Anti-lymphocyte monoclonal antibodies | 8.2.3 |
| Other immunomodulating drugs | 8.2.4 |
| Oestrogens in malignant disease | 8.3.1 |
| Progestogens in malignant disease | 8.3.2 |
| Androgens in malignant disease | 8.3.3 |
| Hormone antagonists in breast cancer | 8.3.4 |
| Other hormone antagonists in malignant disease | 8.3.4 |
| Somatostatin analogues | 8.3.4 |
| **Chapter 9 Nutrition and blood** |  |
| Oral iron | 9.1.1 |
| Parenteral iron | 9.1.1 |
| Drugs used in megaloblastic anaemias | 9.1.2 |
| Drugs used in hypoplastic, haemolytic and renal anaemias | 9.1.3 |
| Drugs used in platelet disorders | 9.1.4 |
| G6PD deficiency | 9.1.5 |
| Drugs used in neutropenia | 9.1.6 |
| Drugs used to mobilise stem cells | 9.1.7 |
| Oral potassium | 9.2.1 |
| Oral sodium | 9.2.1 |
| Oral rehydration | 9.2.1 |
| Oral bicarbonate | 9.2.1 |
| Calcium supplements | 9.5.1 |
| Hypercalcaemia and hypercalcuria | 9.5.1 |
| Magnesium | 9.5.1 |
| Phosphate supplements | 9.5.2 |
| Phospate binding agents | 9.5.2 |
| Fluoride | 9.5.3 |
| Zinc | 9.5.4 |
| Selenium | 9.5.5 |
| Vitamin A | 9.6.1 |
| Vitamin B group | 9.6.2 |
| Vitamin C | 9.6.3 |
| Vitamin D pharmacological | 9.6.4 |
| Vitamin D osteoporosis | 9.6.4 |
| Vitamin E | 9.6.5 |
| Vitamin K | 9.6.6 |
| Drugs used in metabolic disorders | 9.8.1 |
| Acute porphyrias | 9.8.2 |
| **Chapter 10 Musculoskeletal and joint diseases** |  |
| Non-selective NSAIDs | 10.1.1 |
| Cox 2 NSAIDs | 10.1.1 |
| Drugs suppressing rheumatic process - gold | 10.1.3 |
| Drugs suppressing rheumatic process - penicillamine | 10.1.3 |
| Drugs suppressing rheumatic process - antimalarials | 10.1.3 |
| Drugs suppressing rheumatic process - leflunomide | 10.1.3 |
| Drugs suppressing rheumatic process - methotrexate | 10.1.3 |
| Drugs suppressing rheumatic process - other | 10.1.3 |
| Gout and cytotoxic-induced hyperuricaemia | 10.1.4 |
| Other drugs for rheumatic diseases | 10.1.5 |
| Drugs enhancing neuromuscular transmission | 10.2 |
| Skeletal muscle relaxants | 10.2 |
| Enzymes for soft tissue inflammation | 10.3.1 |
| Rubefacients | 10.3.2 |
| Topical NSAIDs | 10.3.2 |
| Capascain | 10.3.2 |
| **Chapter 11 Eye** |  |
| Antibacterial eye preparations | 11.3.1 |
| Antiviral eye preparations | 11.3.3 |
| Corticosteroid eye preparations | 11.4 |
| Other anti-inflammatory eye preparations | 11.4 |
| Mydriatics and cycloplegics | 11.5 |
| Glaucoma beta blockers | 11.6 |
| Glaucoma prostaglandin analogues | 11.6 |
| Glaucoma sympathomimetics | 11.6 |
| Glaucoma carbonic anhydrase inhibitors | 11.6 |
| Glaucoma other | 11.6 |
| Tear deficiency, ocular lubricants and astringents | 11.8.1 |
| **Chapter 12 Ear, nose and oropharynx** |  |
| Otitis externa | 12.1.1 |
| Nasal allergy topical anthistamines and cromoglicate | 12.2.1 |
| Nasal allergy topical corticosteroids | 12.2.1 |
| Topical nasal decongestants | 12.2.2 |
| Nasal preparations for infection | 12.2.3 |
| Drugs for oral ulceration and inflammation | 12.3.1 |
| Oropharyngeal anti-infective drugs | 12.3.2 |
| Treatment of dry mouth - oral drugs | 12.3.5 |
| Treatment of dry mouth – local treatment | 12.3.5 |
| **Chapter 13 Skin** |  |
| Emollients | 13.2.1 |
| Barrier preparations | 13.2.2 |
| Topical local anaesthetics and antipruritics | 13.3 |
| Topical corticosteroids | 13.4 |
| Preparations for eczema | 13.5.1 |
| Topical vitamin D and analogues for psoriasis | 13.5.2 |
| Topical tazarotene for psoriasis | 13.5.2 |
| Topical tars for psoriasis | 13.5.2 |
| Topical dithranol for psoriasis | 13.5.2 |
| Topical salicylic acid for psoriasis | 13.5.2 |
| Oral drugs for psoriasis | 13.5.2 |
| Skin drugs affecting immune response | 13.5.3 |
| Benzoyl peroxide and azelaic acid for acne | 13.6.1 |
| Topical antibacterials for acne | 13.6.1 |
| Topical retinoids for acne | 13.6.1 |
| Other topical preparations for acne | 13.6.1 |
| Oral hormone treatments for acne | 13.6.2 |
| Oral retinoids for acne | 13.6.2 |
| Shampoos and other preparations for scalp and hair conditions | 13.9 |
| Antibacterial skin preparations | 13.10.1 |
| Antifungal skin preparations | 13.10.2 |
| Antiviral skin preparations | 13.10.3 |
| Parasiticidal skin preparations | 13.10.4 |
